# Milk Exosomes: Next-Generation Agents for Delivery of Anticancer Drugs and Therapeutic Nucleic Acids

**DOI:** 10.3390/ijms241210194

**Published:** 2023-06-15

**Authors:** Anna M. Timofeeva, Anastasia P. Paramonik, Sergey S. Sedykh, Georgy A. Nevinsky

**Affiliations:** 1 SB RAS Institute of Chemical Biology and Fundamental Medicine, 630090 Novosibirsk, Russia; anna.m.timofeeva@gmail.com (A.M.T.); paramonik.ap@gmail.com (A.P.P.); nevinsky@niboch.nsc.ru (G.A.N.); 2 Faculty of Natural Sciences, Novosibirsk State University, 630090 Novosibirsk, Russia

**Keywords:** exosomes, milk exosomes, milk vesicles, extracellular vesicles, drug delivery, target delivery, visualization, cargo loading

## Abstract

Exosomes are nanovesicles 40–120 nm in diameter secreted by almost all cell types and providing humoral intercellular interactions. Given the natural origin and high biocompatibility, the potential for loading various anticancer molecules and therapeutic nucleic acids inside, and the surface modification possibility for targeted delivery, exosomes are considered to be a promising means of delivery to cell cultures and experimental animal organisms. Milk is a unique natural source of exosomes available in semi-preparative and preparative quantities. Milk exosomes are highly resistant to the harsh conditions of the gastrointestinal tract. In vitro studies have demonstrated that milk exosomes have an affinity to epithelial cells, are digested by cells by endocytosis mechanism, and can be used for oral delivery. With milk exosome membranes containing hydrophilic and hydrophobic components, exosomes can be loaded with hydrophilic and lipophilic drugs. This review covers a number of scalable protocols for isolating and purifying exosomes from human, cow, and horse milk. Additionally, it considers passive and active methods for drug loading into exosomes, as well as methods for modifying and functionalizing the surface of milk exosomes with specific molecules for more efficient and specific delivery to target cells. In addition, the review considers various approaches to visualize exosomes and determine cellular localization and bio-distribution of loaded drug molecules in tissues. In conclusion, we outline new challenges for studying milk exosomes, a new generation of targeted delivery agents.

## 1. Introduction

Exosomes are membrane vesicles of cellular origin released by cells of various types and found in almost all physiological fluids. Exosomes have a diameter of 40 to 120 nm and are coated with a phospholipid bilayer derived from the membrane of the cell of origin [1,2]. Exosomes have been shown to be involved in intercellular communication and transfer their content from the parent cell to the recipient cells [3,4]. Exosomes are known to contain various biologically important molecules: proteins, lipids, DNA, mRNA, microRNA, and others [3].

Exosomes have been found in blood, urine, saliva, milk, ascites fluid, and other biological fluids [5]. The high biocompatibility of exosomes makes them an attractive alternative tool for the delivery of therapeutically relevant drugs, albeit poorly explored to date [3,6].

The benefits of using exosomes are: (1) low immunogenicity [7]; (2) easy entry into cells due to the natural origin and biocompatible structure allowing for more efficient cargo delivery [8]; (3) robust membrane with a potential to protect therapeutically relevant molecules from degradation [9]; (4) capability for long-term circulation in the body [8]; (5) the potential for their membrane to be artificially modified for targeted delivery, enabling tissue-specific or cell-specific distribution [10]. (6) As for milk exosomes, their advantage is that milk is an inexpensive source of exosomes, compared with culture fluid, and safe, compared with blood and other biological fluids, available on a preparative scale [11]. Moreover, exosomes can cross the blood–brain barrier [12,13] and the placental barrier [14,15]. Their ability to cross the blood–brain barrier can be used to develop delivery systems to brain tissue, for example, in Alzheimer’s and Parkinson’s disease [16].

Exosomes have been found in almost all body fluids [2]. Many researchers use exosomes isolated from cell cultures [17,18], although the yield of exosomes from this source is rather low. Moreover, there is a possibility of contamination by vesicles from the serum used for cell cultivation. Another concern is the safety of exosomes derived from cancer and immortalized cell lines to be used in therapy [19].

Milk is a unique natural source of exosomes available in semi-preparative and preparative quantities. Milk exosomes are highly resistant to the harsh conditions of the gastrointestinal tract, with in vitro studies indicating their affinity for epithelial cells, cellular uptake by the endocytosis mechanism, and, importantly, their potential to be used for oral delivery [20]. Due to their membrane containing hydrophilic and hydrophobic components, milk exosomes can be loaded with hydrophilic and lipophilic drugs [21].

The mechanism of exosome formation involves a stage of endocytosis and release by intraluminal budding of multivesicular bodies with the cell membrane of mammary epithelial cells. Milk exosomes have been reported to contain CD9, CD63, CD81, CD82, HSP70, HSP90, Alix, TSG101, annexin, and Rab GTPases [22,23]. Previously, we described in detail the proteins, lipids, and nucleic acids that make up milk exosomes [11,24]. Various sources have reported hundreds and thousands of proteins and mRNAs in milk exosomes. According to our data on protein content in well-purified exosome preparations from horse milk [25,26], numerous proteins, such as caseins, cannot be intrinsic components of milk exosomes but are co-purified with exosomes by crude purification methods. Thus, insufficiently purified preparations can be credited with at least part of the biological functions of milk exosomes. Thus, further studies should be conducted on highly purified preparations of milk exosomes [11].

Exosomes were first described in human milk in 2007 [27]. Nowadays, milk exosomes are regarded as a promising alternative to exosomes derived from cell cultures, with great potential as drug delivery vehicles [20,28]. One of the advantages of exosomes isolated from milk is the high availability of milk, which no other exosome sources can compete [11]. Most global population regularly consumes significant amounts of milk and dairy products, making dairy exosomes a potentially safe delivery vehicle [29]. Bovine milk exosomes were demonstrated to exhibit low systemic immunogenicity in vivo [20,30]. In addition, milk exosomes were reported to be highly resistant to the harsh conditions of the gastrointestinal tract, capable of crossing biological barriers and reaching peripheral tissues suggesting milk exosomes as promising agents for oral minimally invasive delivery [29,31,32]. The use of milk exosomes is considered for liquid biopsy of breast cancer [33]. An increased expression of TGFβ2 in milk exosomes may be associated with the development of breast cancer [34]. In addition, changes in the expression of microRNA [35] and protein components [36] of milk exosomes may be associated with inflammation of the mammary gland (mastitis) in cows. We believe that the use of milk exosomes as an object for liquid biopsy is of little value for humans due to the relatively low detection rate of breast cancer in lactating women, the absence of troubles with a diagnosis of mammary gland inflammation, and the relatively low duration of lactation [37]. Although it cannot be ruled out, the short lactation period explains, in turn, a small number of publications on the use of milk exosomes for diagnosing breast pathologies.

In the previous review [11], we considered the prospects of using milk exosomes to deliver anticancer drugs. Over the past three years, a number of articles appeared with data on new ways of delivering therapeutic molecules using milk exosomes. However, none of the clinical trials using milk exosomes are registered on clinicaltrials.org to the current date, and only some of the approaches of drug delivery previously tested for exosomes from other sources were tested for milk exosomes. In this review, we have focused on the analysis of the structure of milk exosomes, the advantages of milk exosomes as promising delivery agents of therapeutically relevant molecules, their loading, and possible targeting for more targeted delivery. Here, we discuss and address specific aspects of studies already reported in the literature that we believe to be necessary to initiate the practical application of milk exosomes as next-generation delivery agents in cell culture, model animal organisms, and, potentially, in humans.

## 2. Milk Exosomes—Promising Agents for Oral Delivery

Milk exosomes have several advantages compared to exosomes from other biological fluids, including natural compatibility for [20]. On the one hand, oral delivery is one of the least invasive ways to deliver therapeutically important biomolecules. On the other hand, the aggressive environment of the gastrointestinal tract poses significant problems for their delivery [38,39].

In vitro studies show that milk-derived exosomes have an affinity for intestinal epithelial cells [40,41] and can enter the cells by endocytosis [42,43]. Therapeutic drugs encapsulated in exosomes can be delivered orally into the bloodstream of laboratory animals in a bioactive form [44]. For example, milk exosomes and their microRNAs have been shown to be stable in gastric juice and pancreatic secretion [40,45] and stable when incubated with saliva or bile [41].

Orally administered milk exosomes exhibit a wide bio-distribution, with labeled exosomes found in the liver, spleen, kidneys, pancreas, ovaries, lungs, heart, brain, and colon [29,32,46]. The greatest amount of milk exosomes is accumulated in the liver and spleen due to the phagocytic activity of Kupffer macrophages in these organs [47]. Histological evaluations confirm exosome accumulation does not cause tissue damage or changes in liver tissue structure [48].

**Figure 1:**
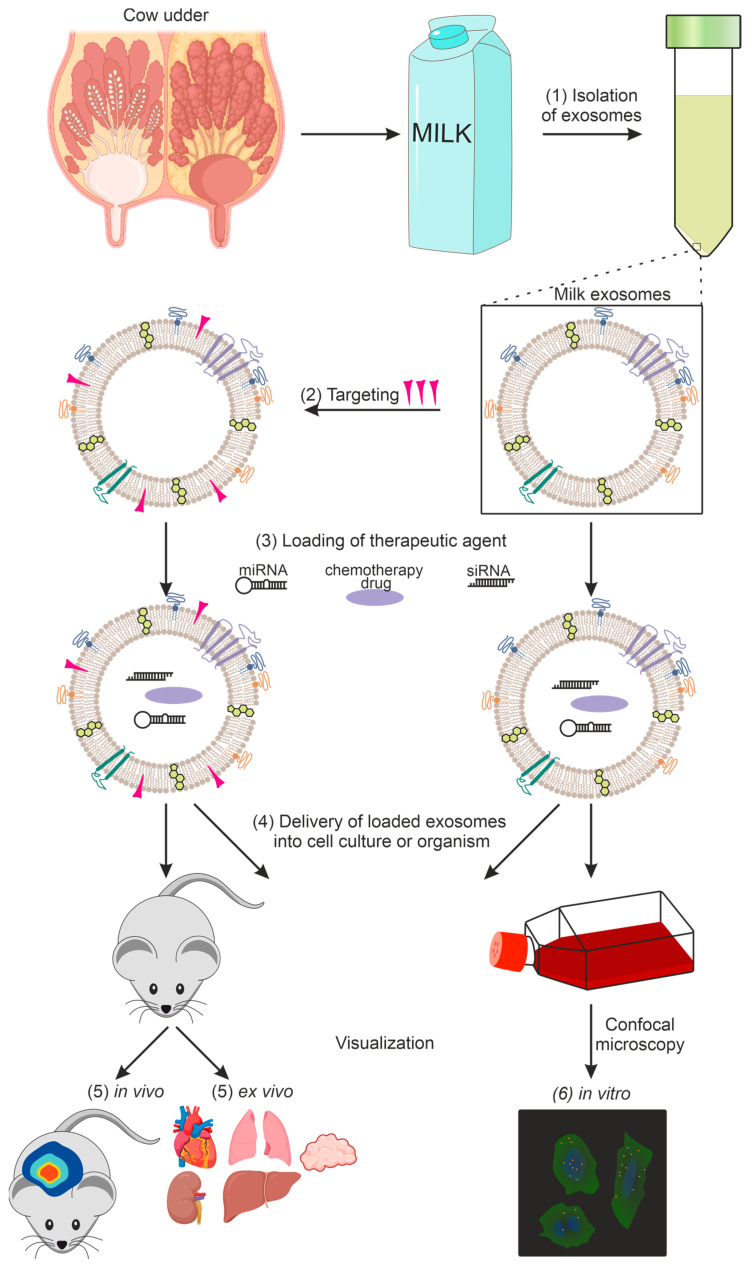
Using exosomes as a delivery vehicle for therapeutic agents: (1) exosome isolation, (2) targeting of exosomes, (3) loading of the therapeutic agent, (4) delivery of loaded exosomes into cell culture or organism, (5) in vivo and ex vivo imaging, (6) in vitro imaging.

The following sections cover the main stages of research on exosomes as drug delivery systems. Section 3 is devoted to the isolation of exosomes from milk, and Section 4 is on their physicochemical characterization. Preparations that can be loaded into lactic exosomes will be discussed in Section 5, and methods for loading them in Section 6. Particular attention will be paid to exosome targeting in Section 7 and exosome imaging in the body and/or in cell culture in Section 8. A general diagram of the use of milk exosomes as a delivery vehicle from isolation to imaging is shown in Figure 1.

Comparison of the bioavailability of radiolabeled goat milk exosomes with different routes of administration shows that the intravenous route of administration led to a rapid accumulation of exosomes in the liver; intraperitoneal administration led to the slow appearance of exosomes in the bloodstream, and their accumulation into the thyroid gland and lungs; intranasal administration led to the entry of exosomes into the brain [49].

## 3. Isolation of Milk Exosomes

Milk contains large amounts of proteins and lipids (including fats). It is necessary to remove these impurities before isolating exosomes, since fat, cell debris, and free proteins significantly hamper the production of pure exosome preparations. Methods for isolating and purifying milk exosomes include differential centrifugation, tangential flow filtration, density gradient centrifugation, ultracentrifugation, immune affinity capture, gel filtration, and other types of chromatography, while microfluidic techniques and some other methods have not yet been used for exosome isolation from milk [50,51]. Standard approaches for isolating and purifying exosomes from milk and other biological fluids are shown in Table 1.

According to MISEV2018, existing exosome isolation methods are classified by recovery and specificity, and no single high recovery and high specificity method have been developed so far. Therefore, a combination of several methods is usually used to isolate pure exosome preparations that do not contain impurities of co-precipitating structures, with the first method being high recovery and the subsequent methods being high specificity ones [57].

### 3.1. Centrifugation

Differential centrifugation is the most commonly used method for isolating and purifying milk exosomes [58]. This method combines low-speed and high-speed centrifugation [59]. Low-speed centrifugation allows cell debris, fat, and some free proteins to be removed from the milk. Centrifugation after titration to pH 4.6 [26,60] and ultracentrifugation can remove a significant amount of free proteins [58]. Unfortunately, ultracentrifugation produces a precipitate that is only a fraction enriched in exosomes and contains a mass of co-precipitated impurity proteins [25,26]. Thus, differential centrifugation is a simple method allowing crude exosome preparation to be obtained using equipment available in various laboratories. The disadvantages of this method are high time costs, numerous proteins co-precipitated with exosomes, and a possibility of disruption of the vesicle structure. Thus, differential centrifugation cannot be considered the optimal method for the preparative production of exosomes. However, this method can be used in combination with other methods.

Density gradient centrifugation relies on different coefficients of sedimentation of vesicles and particles. A density gradient is obtained by layering sucrose or iodixanol solutions in decreasing concentration in a centrifuge tube and placing the sample over the gradient. Centrifugal force enables milk exosomes to be fractionated in the density range of 1.13–1.19 g/mL. Exosomes obtained with this method tend to have high purity, but the steps are labor- and time-consuming [59].

### 3.2. Preliminary Protein Precipitation

Milk contains numerous proteins that make the isolation and purification of exosomes difficult. In particular, the isolation of exosomes from milk is hindered by caseins, the main curdling proteins [61]. There is a universal method of casein removal using isoelectric precipitation. The acidification of pre-defatted bovine milk to pH 4.6 by adding HCl or acetic acid facilitates protein precipitation and improves the subsequent steps of exosome isolation [25,26,62]. It is worth noting that although the acidification with HCl or acetic acid does not change the content of exosomes, it does cause a partial degradation of exosome membrane proteins [63]. A method of preheating, acidification, and centrifugation of milk and subsequent ultrafiltration has been described [30]. Additionally, freezing milk whey at −80 °C before ultracentrifugation has been reported to remove cell debris and apoptotic cells [64].

### 3.3. Immunoaffinity Isolation

The surface of milk exosomes has many specific markers: CD9 [65], CD81 [65], CD63 [21], HSP70 [66], and TSG101 [21]. Methods of immunoaffinity isolation allow exosome preparations to be obtained by the interaction of exosome membrane proteins with antibodies against these proteins [64]. Specific antibodies such as anti-CD9, anti-CD63, and anti-CD81 are used for immunoaffinity purification [25,26].

Antibodies to specific exosome markers can be immobilized on magnetic particles or chromatographic sorbent pellets, as well as in microfluidic devices. Incubation with immobilized antibodies results in specific exosome binding, washing of the nonspecifically sorbed molecules, and further elution of the exosomes. Some studies, such as nucleic acid isolation, trypsinolysis, and further proteomic analysis, can be performed directly with exosomes bound with antibodies on the sorbent, magnetic particles, or other carriers. In some instances, this method can ensure the morphological integrity of exosomes, is highly specific, and is easy enough to use without requiring special equipment. However, affinity chromatography requires a liquid chromatograph. The disadvantages of this method are high cost due to the price of antibodies and low scalability, making it suitable only for obtaining analytical quantities of exosomes [67,68]. In addition, the biological activity of exosomes is affected by the pH of the solution and the salt concentration used in exosome elution, which does not facilitate the subsequent extraction and purification steps and, especially, the use of exosomes for delivery.

### 3.4. Microfluidic Technologies

Microfluidic technologies are based on fractionating by size, density, immunoaffinity, and electrophoretic and electromagnetic methods. These technologies are classified into immune affinity capture, filtration via nanoporous membranes, and exosome capture on porous structures such as nanowires and porous ciliated structures [69].

These technologies have not yet been used for exosome isolation from milk [70], but immunoaffinity capture devices for exosomes based on CD63 binding have been developed for isolation from other biological fluids, for example, [71]. In addition, a ciliated micropillar plate has been developed to selectively capture particles in the 40–100 nm range [72]. Microfluidic methods are advantageous due to the higher fractionation rate than ultracentrifugation.

### 3.5. Use of Precipitation-Based Isolation Kits

Manufacturers’ recommendations state that some commercial exosome extraction kits are suitable for isolating vesicles from milk. For example, exosomes can be obtained by precipitation using polyethylene glycol, and the precipitate can then be isolated by low-speed centrifugation or filtration [73]. These kits are generally easy to use and do not require special expensive equipment. However, the efficiency of isolation and purification of exosomes from milk with the kits available on the market is not always high. Such methods can be complicated by contaminated reagents used for isolation or co-precipitating proteins and impurities [74].

The MISEV 2018 recommendations state that several isolation methods should be used to isolate exosomes. We agree with these recommendations, even though they make isolation very difficult and expensive. A universal one-step method for the isolation of exosome preparations from milk (as well as from other biological fluids) without co-precipitating protein impurities has yet to be proposed and is unlikely to appear in the foreseeable future.

## 4. Exosome Structure Analysis

Of particular importance is analyzing the structure of exosomes isolated from milk. No “gold standard” has yet been proposed to characterize exosomes isolated from milk (and from other biological fluids) in terms of their physical and biological properties. It is possible to analyze exosomes by size, morphology, and membrane proteins. A physical analysis is performed using nanoparticle tracking analysis (NTA), dynamic light scattering (DLS), atomic force, and transmission and scanning electron microscopy. In contrast to other methods, NTA also allows the analysis of particle concentrations to be performed. Biochemical analysis is performed using immunoblotting, flow cytometry, or proteomic analysis [75]. According to the MISEV2018 recommendations, a comprehensive analysis is required: quantitative analysis of vesicles; analysis of several proteins on the surface (non-specific tissue, specific tissue, cytosolic, presented in extracellular vesicles, and absence of “negative marker,” uncharacteristic for vesicles, not to mention the importance to indicate the position of these markers: in the membrane or lumen); characterization of individual vesicles by electron or atomic force microscopy methods [57].

### 4.1. Physical and Immunological Analysis of Exosomes

Flow cytometry [58] is one of the most common methods for analyzing exosome surface antigens, with specific fluorescently labeled antibodies used for antigen analysis. The exosome suspension is shot with a laser beam that scatters as it crosses the exosome, producing a fluorescence signal recorded by a series of detectors. Despite not requiring any special equipment other than a flow cytometer available in many laboratories performing cell culture studies, traditional flow cytometry protocols and most instruments are challenged by the small size of exosomes falling outside the detection limit, around 100 nm. These factors have prompted exosome-specific protocols, such as the initial sorption of exosomes on the surface of latex beads [76,77].

Nanotracking analysis (NTA) involves illuminating a suspension of nanoparticles with a laser beam and determining their position using a dark-field microscope [78]. Although particles smaller than 200 nm are below the resolution limit of light microscopy, the laser-scattered light produced by exosomes can be detected by a digital camera, allowing the vesicle position to be visualized [79]. Since exosomes and other vesicles in suspension are characterized by Brownian motion, NTA tracks their motion in real time and measures their average velocity, which, depending on their size, can be used to calculate their diameter [80]. NTA is widely used to estimate the exosome size and concentration in solution rapidly [79]. NTA can detect exosomes in the range of 50–250 nm, which exceeds the detection limit of flow cytometry and allows for plotting the exosome diameter distribution [79].

The dynamic light scattering (DLS) method determines the size of nanoparticles, extracellular vesicles, and exosomes: it measures the hydrodynamic particle size by scattering light from a laser that passes through a solution and analyzes the modulation of the scattered light intensity as a function of time. Brownian motion of particles correlates with their hydrodynamic diameter: the smaller the particle, the faster it diffuses. The DLS device calculates a correlation function mathematically related to the particle size and its time-dependent light scattering capacity [81,82]. DLS uses two parameters characterizing particle size distribution: average hydrodynamic particle size (Z-average) and polydispersity index (PDI), a dimensionless value characterizing the distribution width. Aggregated particles have a high polydispersity index and a large scatter of particle sizes. A significant disadvantage of this method is the difficulty in distinguishing objects that differ in diameter by less than a factor of two [83].

Transmission electron microscopy (TEM) and atomic force microscopy (AFM) allows exosomes to be visualized and their size to be determined. TEM involves fixing exosomes and layering them on an electron microscopy grid coated with a carbon/formvar film, followed by staining with uranyl acetate. Visualization in TEM is performed using a transmission electron microscope [84] equipped with a camera. One variant of this method is gold immune staining, enabling visualization of exosome surface proteins using specific monoclonal antibodies [85] and gold nanoparticles. Measurement using AFM is in general faster than TEM; however, this method does not allow the use of immunochemical approaches.

**Figure 2:**
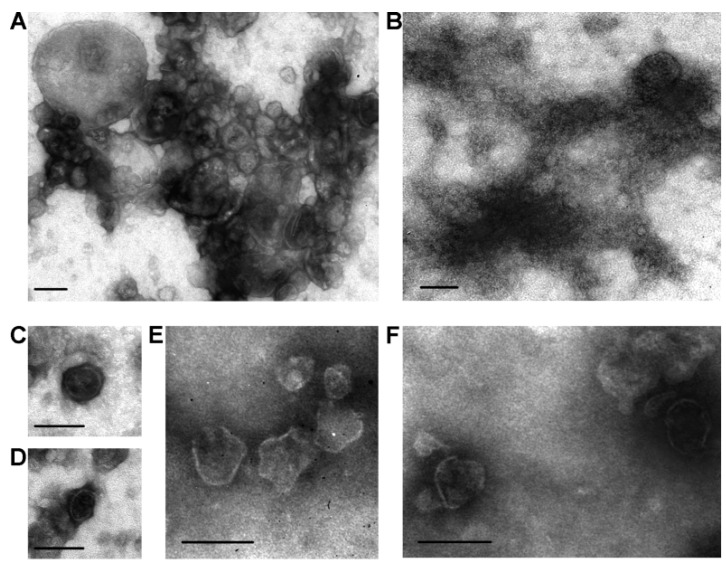
Transmission electron microscopy analysis of horse milk exosomes: preparations of vesicles after different purification stages. A preparation obtained from horse milk after centrifugation and ultracentrifugation (**A**,**B**). Amid the visible structures, vesicles of 40–100 and over 100 nm in size are found, which can be attributed to various vesicles, including exosomes. Filtration of exosome preparations through a 100 nm filter removes any structure larger than 100 nm, with the relative content of amorphous material corresponding to impurity proteins being significantly reduced (**C**,**D**). Upon gel filtration, exosome preparations contain no visible protein amorphous material (**E**,**F**). The data are taken from [60]. Scale bar corresponds to 100 nm.

We were the first to isolate horse milk exosomes containing CD9, CD63, and CD81 in [60]. Exosome preparations were obtained using the standard centrifugation and ultracentrifugation methods described earlier. The exosomes had characteristic morphology and shapes and were 40–100 nm in size (Figure 2A,B). Ultrafiltration of crude exosome preparations through a 0.1 μm filter resulted in particles >100 nm and large protein associates being removed (Figure 2C,D). For most preparations, exosomes were most effectively separated from impurity proteins using an additional purification step, gel filtration (Figure 2E,F). In subsequent studies, we have described sub-fractions of horse milk exosomes containing two tetraspanins simultaneously: CD9 and CD63 [25], and CD9 and CD81 [26].

**Figure 3:**
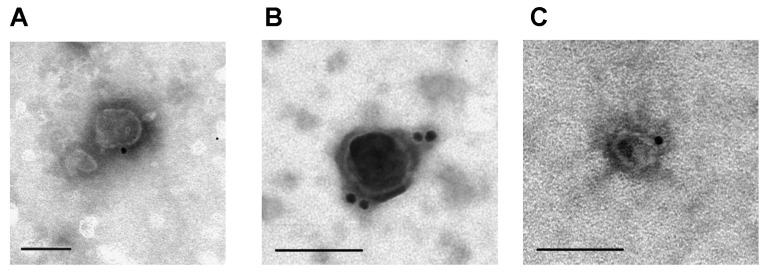
Analysis of horse milk exosomes isolated by affinity chromatography on anti-CD9, CD63, and CD81-Sepharose labeled with monoclonal antibodies against CD9 (**A**), CD63 (**B**), and CD81 (**C**) bound to gold nanoparticles. The data are taken from [25,26]. Scale bar corresponds to 100 nm.

Immunoaffinity labeling and transmission electron microscopy are widely used for visualizing tetraspanins on the surface of milk exosomes. Figure 3 shows exosome preparations isolated by affinity chromatography on sorbents containing polyclonal antibodies against CD9, CD63, and CD81 and labeled with monoclonal antibodies against these antigens: CD9 (Figure 3A), CD63 (Figure 3B), and CD81 (Figure 3C). Thoroughly purified milk vesicles satisfy typical requirements for exosome morphology, size, and surface tetraspanin content.

### 4.2. Biochemical Analysis of Exosomes

The protein composition of exosomes is analyzed by one- or two-dimensional electrophoresis in polyacrylamide gel (SDS-PAGE) followed by protein staining, immunoblotting, or proteomic analysis (MALDI TOF MS or ESI TOF MS) [86], with an exosome biomarker database collected at www.exocarta.org.

The most commonly used exosome markers, including those described on the surface of milk exosomes, are CD9 [66], CD63 [21], and CD81 [65] tetraspanins [87]. The detection of these tetraspanins by Western blotting and immune gold staining is widely used to confirm exosome content in a sample [88,89].

Various articles reported milk exosome preparations containing from tens to hundreds or thousands of proteins. Poorly purified exosome preparations were found to contain such allergenic proteins as beta-lactoglobulin and caseins, while the preparations after affinity chromatography and gel filtration contained only about a dozen basic proteins, with no allergens detected [25]. Thus, thoroughly purifying milk exosome preparations is essential for them to be used as a delivery vehicle.

## 5. Delivery of Therapeutically Relevant Drugs by Milk Exosomes

In the following, we discuss the therapeutically relevant drugs already used for loading into milk exosomes and subsequent delivery to cells.

### 5.1. Small Interfering RNAs

Small interfering RNAs (siRNAs), double-stranded RNA molecules with a length of 19 to 25 nucleotides triggering a specific target mRNA knockdown, are considered to be promising tools for specific gene silencing. Their advantages are efficiency, a small number of side effects, and ease of siRNA synthesis [90,91]. Due to the difficulty in cellular uptake of naked siRNA [90,91] and its rapid degradation by plasma nucleases in the systemic bloodstream [12], it is necessary to encapsulate siRNA in a carrier for efficient delivery and uptake by target cells. Currently, Patisiran, one of the therapeutic siRNA drugs approved for therapeutic use, is packaged in lipid nanoparticles. A number of studies suggest the efficiency of using dairy exosomes to deliver siRNA, using such exosome loading methods as electroporation and chemical transfection. siRNAs loaded into exosomes are resistant to RNase and are taken up by cancer cells, suppressing the expression of cancer target genes in vitro. Bovine milk exosomes have been shown to be able to deliver siRNAs that specifically turn off *VEGF*, *EGFR*, *AKT*, *MAPK*, *KRAS*, *SUR*, *BCL2* [92], *Keap1* [93] genes, and other therapeutically relevant [94] and target genes [94].

### 5.2. MicroRNAs

MicroRNAs are a class of short, evolutionarily conserved, non-coding RNAs of 20–24 nucleotides in length [95]. Among molecules loaded into exosomes, microRNAs attract particular attention since they have been reported to regulate the expression of 30 to 60% of human genes, and the loss of microRNA biogenesis in mice with Dicer protein knockout is lethal for the embryo [96,97]. The fact that microRNAs regulate gene expression at the posttranscriptional level [98] is used to develop new therapeutic agents. However, despite the high therapeutic potential of microRNAs, their usage is hampered by many obstacles. The negative charge of microRNAs makes it difficult for them to pass through cell membranes [99]. In addition, miRNAs are unstable in vitro and easily degraded [100].

Exosomes have been shown to increase microRNA stability by protecting them from RNase degradation. However, not only can microRNAs be loaded inside the exosome, but they can also remain bound to the exosome membrane outside, with RNase A treatment leading to microRNA degradation. Thus, a degree of microRNA internalization resulting from loading can be judged by the degree of microRNA degradation [101].

Several publications are devoted to using milk exosomes as delivery vehicles for synthetic microRNAs. For example, loading miR-148a-3p into milk exosomes resulted in a statistically significant increase in the concentration of this microRNA in HepG2 and Caco-2 cell lines and led to decreased expression of *AKR1C1*, *AKR1C2*, *CYP3A5*, *CAB39L*, *ODAM*, and *NEGR1* genes [101].

In another study, miR-31-5p was encapsulated in milk exosomes, and the effect on wound healing in diabetic mice was examined. miR-31-5p was observed to accelerate diabetic wound healing by improving endothelial cell function and stimulating angiogenesis [102].

Thus, synthetic microRNAs delivered by milk exosomes are functional in recipient cells and alter gene expression in cells [101].

### 5.3. Therapeutic and Chemotherapeutic Drugs

Milk exosomes can be loaded with therapeutic and chemotherapeutic drugs [92,101,103]. Loading bioactive substances into milk exosomes has been shown to increase their circulation time in the bloodstream, therapeutic activity, and bioavailability [20].

#### 5.3.1. Curcumin and Celastrol

Curcumin is a natural molecule possessing a number of therapeutic properties, but its pharmaceutical use is limited due to its poor solubility in water and low systemic bioavailability. Curcumin, internalized in bovine milk exosomes, demonstrates increased stability compared to free curcumin. Curcumin encapsulated in milk exosomes has increased intestinal permeability [104], and a threefold increase in the transport of curcumin loaded into milk exosomes in the transepithelial monolayer of Caco2 cells [104].

Celastrol is a triterpenoid of plant origin and a known inhibitor of Hsp90 and NF-κB activation pathways. Celastrol has been demonstrated to inhibit the proliferation of various cancer cells in vitro and in vivo [105,106]. Exosomes can be loaded with celastrol in the presence of ethanol (<10%) by simple mixing at room temperature, with a drug loading efficiency exceeding 20%. The size and PDI of celastrol-loaded bovine milk exosomes are virtually unchanged [107].

#### 5.3.2. Paclitaxel

Paclitaxel is widely used as a first- or second-line chemotherapeutic agent to treat a wide range of human cancers, including lung cancer. This drug exhibits antiproliferative and apoptotic effects against cancer cells resulting from binding to β-subunits of tubulin, blocking polymerization of microtubules in the cell, and preventing cell division [108]. Despite rather high efficacy, paclitaxel has limited application due to low water solubility and high toxicity. Paclitaxel encapsulated in milk exosomes has been shown to exhibit superior action against breast (T47D and MDA-MB-231) and lung (A549 and H1299) cancer cells [20].

Paclitaxel-loaded bovine milk exosomes were reported to effectively inhibit tumor growth in a subcutaneous lung cancer model in mice, with the paclitaxel-loaded exosome preparation being twice more effective when administered orally than when administered intraperitoneally [109]. Paclitaxel drug loaded in exosomes and delivered orally demonstrated significant inhibition of lung tumor xenografts without causing systemic toxicity or immunotoxicity compared to intravenous paclitaxel administration [108].

Paclitaxel loaded into exosomes was demonstrated to inhibit tumor growth by 60%, while free paclitaxel only by 31%. This result may be due to the prolonged release of the drug and the significantly lower systemic and immunologic toxicity of exosome-bound paclitaxel [109].

#### 5.3.3. Doxorubicin

Doxorubicin is one of the most effective chemotherapeutic drugs used in clinical practice to treat a wide range of cancers. The doxorubicin functioning mechanism is its incorporation into the double helix of DNA, inhibiting the action of topoisomerase II and blocking DNA replication [110]. The use of doxorubicin is limited because of the high risk of cardiotoxicity [111], the risk of pericarditis, heart failure, and arrhythmia.

A modification of bovine milk exosomes with two ligands, neuropilin receptor agonist peptide and hypoxia-responsive lipid was used to deliver doxorubicin to triple-negative breast cancer cells. These modifications caused cytostatic release under tumor hypoxia, and the antitumor effect was confirmed in the 3D spheroid model [112].

A system of doxorubicin delivery with pH- and light-sensitive effects was also designed based on milk exosomes. This construct was demonstrated to have high antitumor activity against oral squamous cell carcinoma on HSC-3, SCC-9, and CAL-27 cell lines [113].

#### 5.3.4. Antioxidants

Cow milk exosomes exhibit antioxidant properties, with their addition leading to an increase in glutathione peroxidase and superoxide dismutase activity in IEC-6 cell culture and a decrease in the concentration of reactive oxygen species after treatment of these cells with hydrogen peroxide [114]. The antioxidants astaxanthin, α-tocopherol, chlorogenic acid, β-carotene, rutin, and resveratrol were loaded into cow milk exosomes by incubation and tested for biological activity on MDA-MB-231 (human breast cancer) cell line, with antioxidant-encapsulated exosomes retaining their antioxidant properties for up to 3 weeks and exhibiting no cytotoxicity [115].

## 6. Loading of Functional and Biologically Active Molecules into Exosomes

**Figure 4:**
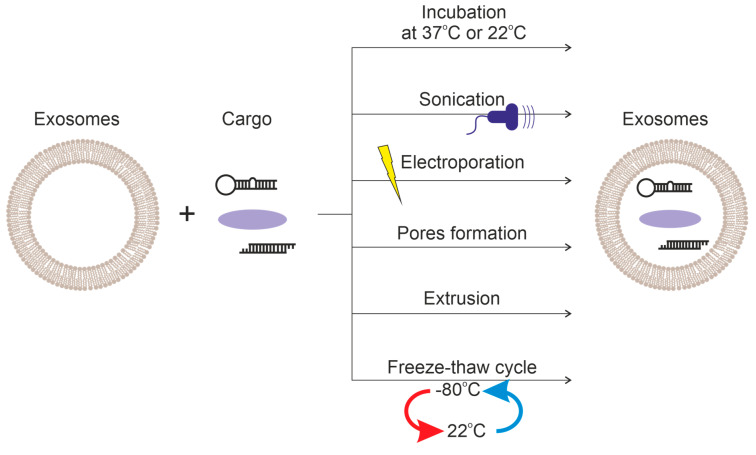
Comparison of methods of loading cargo into exosomes.

Exosomes are composed of hydrophilic and hydrophobic components. Therefore, they can serve as a delivery vehicle for both hydrophilic and lipophilic molecules. Various methods are employed to load drugs into exosomes: incubation (passive), and active [116], ultrasound treatment [21], extrusion [116], freeze-thaw cycles [21], electroporation [116], and treatment with saponins [102]. The loading principles of these methods are summarized in Figure 4.

### 6.1. Incubation

Exosomes are incubated with the loaded drug solution, usually at room temperature for 90 min to 1–2 days, resulting in the diffusion of biologically relevant molecules into the exosomes along a concentration gradient. The efficiency of this loading method depends on the hydrophobicity of the loaded molecules. Hydrophobic drugs easily penetrate the lipid membrane of exosomes [117]. The rate of drug diffusion depends on its physicochemical properties. For example, when incubated with exosomes of paclitaxel and doxorubicin at 37 °C for 2 h, doxorubicin demonstrated greater loading efficiency compared to paclitaxel [118].

The incubation method was used to load curcumin, celastrol, anthocyans, microRNA, and vitaferin A into milk exosomes. The resulting preparations were tested in vivo for cancer treatment in mice [92,119]. Experiments on loading vitaferin A, curcumin, paclitaxel, and docetaxel into milk exosomes demonstrated positive results when mixing a drug solution in ethanol or a mixture of ethanol and acetonitrile (1:1 *v*/*v*) with an exosome suspension. The organic solvent used for loading was shown not to affect the structure and stability of the exosomes [119].

The incubation method is simple and inexpensive compared to other methods but inefficient for loading hydrophilic therapeutic molecules and nucleic acids and proteins.

### 6.2. Extrusion

During extrusion loading, exosomes are mixed with a therapeutic drug solution and loaded into a lipid extruder, designed as a syringe with porous membranes 100–400 nm in diameter. Extrusion causes the exosome membrane to rupture and its contents to mix with the drug. Since mechanical forces act on exosome membranes during extrusion, some membrane properties, such as zeta potential and protein structure, can be altered. One article describes such a method of loading the peptide drug Liraglutide into dairy exosomes, with the use of extrusion being significantly more effective than five other loading methods [120].

### 6.3. Electroporation

Electroporation is a commonly used method for loading hydrophilic drugs, including siRNA and microRNA, into exosomes [91]. Exosomes are suspended in an electroporation buffer (with low ionic strength), and an electric field is applied briefly and pulsed. The electric current disrupts the phospholipid membrane of the exosomes, resulting in temporary pore formation. The drug being loaded diffuses inside the exosomes through these pores. Shortly afterward, the integrity of the exosome membrane is restored. The disadvantage of this method is the aggregation of exosomes [121] and a rather low efficiency compared to loading with transfection reagents [94]. Exosome aggregation and precipitation can be reduced by using membrane stabilizers [122,123]. However, using stabilizers is not recommended due to their possible attachment to the exosome membrane. Electroporation was used for the loading of miRNA [102] and siRNA in bovine milk exosomes [94], but in the latter paper, it has not been as effective as chemical loading.

### 6.4. Chemical Transfection

Liposomes, commercially available under the brand names Exo-Fect, lipofectamine, and others, are used for chemical transfection. The chemical transfection reagent is incubated with, for example, siRNA and then with milk exosomes. Such chemical transfection has been shown to increase siRNA loading into milk exosomes several times over the electroporation method [92].

The lipofectamine reagent contains cationic lipids that form liposomes in an aqueous medium. Cationic liposomes can bind negatively charged nucleic acid molecules and help them overcome the electrostatic repulsion of lipid membranes [101]. When lipofectamine is used as a reagent for chemical transfection, the estimated loading efficiency is up to 58%, with no change in the zeta potential of lactic exosomes, indicating that most of the siRNA is incorporated inside the exosomes and that siRNA adhesion to the exosome surface is minimal [94].

### 6.5. Ultrasound

Ultrasound treatment is a physical method applying a mechanical shear force with an ultrasound probe and disrupting the exosome membrane integrity and allowing molecules to penetrate the exosome. Ultrasound treatment is known to decrease the microviscosity of the exosomal membrane significantly. However, the membrane deformation does not significantly affect the membrane-bound proteins or the lipid composition of the exosomes. Following ultrasound treatment, the membrane recovers within 30–60 min at 37 °C [124]. In some cases, however, drugs are not only encapsulated within the exosomes but remain attached to the outer layer of the membrane, resulting in two phases of drug release. The first phase of “explosive” release results from the dissociation of the drug from the exosome surface, followed by a slow release of the drug encapsulated inside the exosome vesicles [125].

The ultrasound treatment method is considered one of the best for loading large therapeutically relevant drugs into exosomes. However, no publications devoted specifically to loading lactic exosomes using this method have been published so far. The disadvantage of the method is that exosomes can lose their integrity due to mechanical damage.

### 6.6. Freezing and Thawing Cycles

In the freeze–thaw cycle method, preparations are incubated with exosomes at room temperature for a fixed period of time, and then the mixture is quickly frozen at −80 or in liquid nitrogen, followed by thawing at room temperature. This process is repeated at least three times to encapsulate the drug. A disadvantage of this method is the aggregation of exosomes, which apparently occurs during freezing and results in a change in the size distribution of exosomes loaded with the drug. Interestingly, this method is also used to fuse exosome and liposome membranes and create particles that mimic exosomes [126]. This method has also not been used previously for loading milk exosomes.

### 6.7. Incubation with Membrane Permeabilizers

Saponin is a surfactant that can form complexes with cholesterol in cell membranes, resulting in pore formation and increased membrane permeability [127]. Saponin can promote the loading of hydrophilic molecules into exosomes [128]. However, there are concerns about the hemolytic activity of saponin in vivo [127]. Hence, exosomes should be purified after incubation with saponin. This method has not been used for loading into milk exosomes.

### 6.8. Comparison of Different Methods of Loading into Exosomes

The different approaches for loading cargo into exosomes have different loading capabilities depending on cargo properties, such as hydrophilicity, hydrophobicity, and molecular weight. However, loading efficiency is only one of the factors to be considered. The integrity and stability of the exosome membrane are also crucial for delivering a therapeutically relevant drug. Table 2 compares the advantages and disadvantages of the above-described loading methods.

## 7. Exosome Targeting

One disadvantage of exosome delivery is that these vesicles can move freely in the extracellular space and biological fluids and are randomly internalized into acceptor cells by diffusion. Their staining with, for example, DiR dye [129] is used to observe the bio-distribution of exosomes. Exosomes have been observed to accumulate in the liver, spleen, kidneys, pancreas, and other organs, indicating uncontrolled bio-distribution of exosomes in vivo. After intravenous injection, lactic exosome concentrations in the liver and spleen reach a maximum after three hours and then decrease. In contrast, when exosomes are administered orally, their concentration in the liver reaches a maximum after 24 h, and no signal is detected after 48 h [32].

A number of studies have shown milk exosomes to have excellent biocompatibility and low toxicity, especially when administered orally [29,130]. However, accurate and targeted drug delivery to exosomes requires modification of their surface, the principles of which are discussed in this section.

### 7.1. Functionalization of the Exosome Surface by Ligands

Folate receptors are present at low levels in normal tissues but are overexpressed in non-small cell lung cancer cells and lung adenocarcinoma cells [131]. More than 100-fold overexpression of folic acid receptors has been demonstrated in lung cancer cells compared to normal tissue [108]. The limited expression and distribution of this receptor make it attractive for targeting lung tumors [132]. Milk exosome conjugates with folic acid have been used to target tumor sites [92].

Human milk exosomes functionalized with folic acid manifest higher accumulation in tumor tissue than folic acid-free milk exosomes and significantly inhibit tumor growth in vivo [133]. Bovine milk exosomes were functionalized with folic acid and then loaded with apherin A, a therapeutic drug of plant origin. Exosomes carrying folic acid on their surface exhibited greater inhibition of tumor growth compared to loaded exosomes without surface functionalization [20]. Paclitaxel was loaded into colostrum exosomes covalently modified with folic acid. As a result, this combination was shown to be cytotoxic against cancer cells and to have no general, systemic, or immunotoxicity in wild-type mice. Covalent binding of folic acid via EDC/NHS chemistry to amino groups found on the surface of exosomes resulted in a significant reduction in tumor size [108].

Hyaluronic acid receptor CD44 is known to be overexpressed in pancreatic, lung, ovarian, and breast cancers [134]. Hyaluronic acid is a specific CD44 ligand: DSPE-PEG 200 and hyaluronan were covalently conjugated to exosome membranes for cancer targeting therapy [135]. Milk exosomes, the surface of which was modified with hyaluronic acid and loaded with doxorubicin, selectively delivered doxorubicin to cancer cells over-expressing CD44 and exhibited increased antitumor activity.

### 7.2. pH and Light-Sensitive Carrier

Physicochemical properties of tissues and cells play an important role in exosome targeting. For example, increased glycolysis resulting in increased lactate concentration and decreased pH in the cytoplasm of tumor cells makes pH-sensitive drugs a convenient targeting tool. A pH- and light-sensitive carrier has been developed to target acidic and hypoxic tumor microenvironments based on milk exosomes [113]. Most solid tumors are known to have a pH of 6.5 to 7.4, making nanoparticles responsive to pH [136]. The imine bond is readily degraded in an acidic solution at a pH below 6.8 [137]. Thus, provided that the exosome membrane is conjugated to hydrazone-bound chemotherapeutic drugs, the cleavage of the imine bond caused by the acidic tumor microenvironment leads to a rapid release of the drug at the tumor site [113].

### 7.3. Introduction of Functional Molecules

Milk exosome membrane engineering has not been described in any article to date. However, approaches for introducing functional molecules onto their surface have been described for exosomes from other sources. This subsection discusses these approaches in terms of their potential application to milk exosomes. Two main approaches for introducing functional molecules to the exosome surface have been described: (1) conjugation with native exosome surface proteins by click chemistry methods [138,139], (2) lipophilic insertion of peptides or proteins onto the exosome membrane surface via a hydrophobic anchor [18,140].

#### 7.3.1. Conjugation with Proteins by Click Chemistry Methods

The exosome surface can be modified by a wide range of ligands using chemical methods. Conjugation can result in the covalent modification of exosome surface proteins, but the efficiency of the modification reaction can be quite low due to the extremely complex surface of milk exosomes.

Click chemistry was used to develop a method for conjugating ligands to the exosome surface. Copper-catalyzed cycloaddition of azide–alkynes (click chemistry) proves to be ideal for the bioconjugation of small molecules and macromolecules on the exosome surface because of its fast reaction time, high specificity, and compatibility in aqueous buffers. Such conjugation does not significantly affect the size of exosomes, nor does it cause any changes in the attachment and internalization of exosomes into recipient cells. The amino groups of exosomal proteins can be easily modified with alkine groups, and then alkine-labeled exosomal proteins can be bound to azide-containing reagents via azide–alkyne cycloaddition reactions. This approach has been used to modify the surface of exosomes with low molecular weight dyes and larger azide-containing proteins [139].

Modified exosomes have been shown to target the area of ischemic brain damage (in an animal model of mouse stroke) and penetrate microglia, neurons, and astrocytes [138]. Chemical modifications can also be used to conjugate large biomolecules into exosomes. Most tumors contain the surface receptor CD47 that interacts with the signal-regulating protein α (SIRPα) on phagocytes and reduces the ability of macrophages to phagocytize tumor cells. Exosomes with SIRPα on their surface have been constructed capable of preventing CD47-SIRPα interaction between cancer cells and macrophages, causing tumor growth to regress [138].

Nevertheless, exosome surface click chemistry for target cell delivery is a relatively new direction. Effective exosome targeting through surface modification is non-trivial and requires further research, especially in terms of the potential clinical use of modified exosomes.

#### 7.3.2. Lipophilic Insertion via Hydrophobic Anchor

Another strategy for chemical modification is the insertion of amphipathic molecules into the lipid bilayer of exosomes. For example, 1,2-dioleoyl-SN cross-linked with polyethylene glycol (PEG)-glycero-3-phosphoethanolamine (DSPE-PEG) can be incorporated into the exosome membrane. It should be noted that DSPE-PEG is FDA-approved for medical use.

Sigma receptors are overexpressed in lung cancer and can act as receptors for targeted exosome delivery. Anisamides, including aminoethylanisamide, are sigma-selective ligands. Aminoethylanisamide bound to DSPE-PEG was successfully conjugated to the exosome membrane [141]. Exosomes modified with aminoethylanisamide demonstrated increased uptake in lung tumor cell lines. Thus, exosomes modified with DSPE-PEG-based ligands hold promise as drug carriers for drug delivery to the tumor [142].

The surface of exosomes can be modified with choline conjugated to RNA aptamers (e.g., against PSMA or EGFR) or with folic acid [143]. Such modified exosomes target siRNAs and microRNAs to the respective tumor sites and increase the antitumor efficacy of the aptamers.

Another approach is to incorporate conjugates with streptavidin into the exosome surface to yield a modular platform where any biotinylated molecule can be specifically attached to the exosome surface. For example, glycerol–phospholipid–PEG (DMPE-PEG) containing streptavidin is used for this purpose. DMPE-PEG provides incorporation into the lipid bilayer of the vesicle membrane [144,145]. In [18], the bio-distribution of biotinylated fluorophores conjugated on the surface of exosomes in the animal body was analyzed. This method can use biotinylated antibodies or peptides that provide targeting to tissues.

## 8. Visualization of Exosomes

Imaging technology is widely used to determine cellular localization and distribution, tissue accumulation and bio-distribution, and pharmacokinetics of drug molecules. Effective imaging of exosomes allows one to describe their intracellular mechanism of action and delivery to different compartments of the cell. Visualization of exosomes is performed on animal models in vivo and ex vivo, as well as on tumor cell cultures in vitro, for example, by confocal microscopy.

Various fluorochromes can penetrate lipid membranes, suggesting their use for determining the membrane structures of exosomes. Several lipophilic dyes based on aminopyriles are used for this purpose, such as FM1-43, FM4-64, MDY-64, DiI, DiO, DiD, DiA, and DiR. Currently, the use of fluorescent dyes is considered the gold standard for exosome imaging. Integration of lipophilic fluorophores into the exosomal membrane is the most widely used labeling method due to its simplicity and low cost [146]. Nevertheless, there are some drawbacks limiting the use of labeled exosomes, such as exosome aggregation and possible dissociation of fluorophores from the exosome membrane, leading to dye release from the exosome into non-target cells and tissues [147]. Given that the size of exosomes is much smaller than the resolution limit of flow cytometers, exosomes are usually sorbed on latex balls before analysis and/or fluorophore staining [148].

Current studies use in vivo imaging systems to document the bioluminescence and fluorescence of small laboratory animals in a non-invasive manner. Imaging systems permit performing fluorescence tomography, three-dimensional reconstruction, and quantitative analysis of targets in deep tissues simultaneously on several anesthetized animals.

Studies of the bio-distribution of milk exosomes labeled with lipophilic dyes have been described in the literature. For example, milk exosomes were labeled with the fluorescent dye DiR and injected once in animals at a dose of 25 mg/kg. Mice and their organs ex vivo were imaged with IVIS Lumina XR-III (Caliper Life Sciences). In this study, exosomes isolated from whole milk showed maximum delivery efficiency [46]. Another study involved injecting 100 μL of DiR-labeled exosomes (60 mg/kg exosome protein) once into mice, and after 4 days, the organs were harvested and imaged ex vivo using Photon Imager Optima (Biospace lab, Paris, France) [20]. When administered intravenously, labeled exosomes accumulated mainly in the liver, and when administered orally, they accumulated quite uniformly in internal organs.

Confocal microscopy is used to confirm whether the cargo-loaded lactic exosome can penetrate tumor cell cultures. For staining actin microfilaments in cells, a solution of phalloidin conjugate with Alexa Fluor 488 [20] is used, for example. Additionally, DNA is stained with DAPI and the cytoskeleton with FITC-phalloidin [102]. Visualization of milk exosomes labeled with other fluorophores makes it possible to suggest the internalization of exosomes inside the cells.

Table 3 summarizes the results of loading lactic exosomes with various drugs and their bio-distribution studies.

## 9. Conclusions

Milk exosomes are promising vehicles for delivering antitumor drugs and therapeutic nucleic acids. A variety of surface functionalization methods to increase the efficiency and targeting of delivery have been shown for exosomes obtained from various sources, mainly cell cultures; for milk exosomes, with only some of these methods being tested so far. A similar situation is observed for loading cargo into milk exosomes.

Compared to other sources, milk is a biological fluid that is available in preparative volumes (for example, tens of liters in the case of cow’s milk), has a low cost (for example, compared to culture fluid), and does not contain viral pathogens harmful for human (for example, compared to blood plasma). To use the full therapeutic potential of milk exosomes, an important issue is the standardization of the conditions for the isolation of exosomes from milk, as well as their additional purification from co-releasing impurities. Unfortunately, such recommendations have not yet been developed.

Further studies should be aimed at analyzing all possible combinations of loading and surface modification of milk exosomes because milk is a unique source of natural exosomes not derived from tumor cells. In this review, we have traced the exosomes’ pathway from their isolation from milk to the visualization of loaded exosomes in animal organs and tissues. It is beyond doubt that these extracellular milk vesicles are next-generation delivery vehicles.

## Figures and Tables

**Table 1:** Extraction and purification of exosomes from milk and other biological fluids: advantages and disadvantages.

Methods of Isolation	Principle of Isolation	Advantages	Disadvantages
Centrifugation [52,53]	Differential centrifugation, density gradient centrifugation, isoelectric deposition	Method simplicity, high yield	Time-consuming, low purity, exosomes can be destroyed, high cost of ultracentrifuges
Ultrafiltration [54]	By vesicle size	Low cost, no expensive equipment required	Low purity
Immunological [25,26]	Binding of antibodies to specific exosome markers	High purity, high specificity, low equipment cost, easy to operate	High reagent cost, low yield
Microfluidic devices [55]	Size, density, surface antigens	High purity, portability, easy to operate, time-saving, automation, speed of extraction	Small sample volume, no method validation Small sample volume, no method validation
Chromatography [52]	Gel filtration, affinity chromatography	High purity, scalability	Requires special equipment, sorbents
Co-precipitation [56]	Precipitation with commercial reagents	Easy to use, time-saving	Expensive, low purity, small sample volume, difficult to scale

**Table 2:** Advantages and disadvantages of different drug loading methods.

Method	Mechanism	Advantages	Disadvantages
Incubation	Diffusion by a concentration gradient	Easy, inexpensive, does not affect exosome integrity	Low capacity of drug loading
Extrusion	Membrane degradation and subsequent recovery	An industrially acceptable loading method	Modification of exosomal membrane properties
Electroporation	High voltage electrical charge produces temporary pores on the exosomal membrane	Effective for loading hydrophilic drugs, siRNA, and microRNA	Possible siRNA precipitation and vesicle aggregation or fusion
Chemical transfection	Requires specific reagents	High loading capacity	Suitable not for all types of molecules
Ultrasound	Decrease in microviscosity of the exosomal membrane	Does not affect membrane proteins and lipids	Disruption of membrane integrity
Freeze–thaw cycles	Deformation of exosome membranes resulting in drug entrapment	An industrially acceptable loading method	Exosome aggregation
Incubation with membrane permeabilizers	Selectively forms a complex with cholesterol to form a porous structure on the membrane surface	High loading capacity	Detergents, such as saponin, may cause hemolysis in vivo

**Table 3:** Loading of lactic exosomes with drugs and bio-distribution of loaded exosomes in mice.

Drug	Loading Method	Mouse Model or Cell Culture	Biodistribution Study	Reference
Chemopreventive (vitaferin A, curcumin) and chemotherapeutic drugs (paclitaxel and docetaxel)	Mixing the drug dissolved in ethanol or 1:1 ethanol/acetonitrile mixture with an exosome suspension in a 1:9 ratio at RT	Female thymus-deprived mice	DiR-labeled milk exosomes administered via probe or intravenous injection. Organs imaged ex vivo using Photon Imager Optima	[20]
Cisplatin	Mixing the preparation dissolved in a 1:1 acetonitrile: ethanol mixture with milk exosome dispersion in a 1:9 ratio at RT	A mouse model of a cancer xenograft derived from A2780CP cells. Intravenous injection	Milk exosomes labeled with PKH26, A2780CP cells labeled with a green actin tracker	[149]
Insulin	Ultrasound treatment	Mouse model of diabetes, oral administration	Glucose measurement	[150]
Curcumin and Resveratrol	Passive incubation, ultrasound treatment, and electroporation	Intravenous injection into the tail vein of mice	Blood and breast tissue were analyzed using UPLC-ESI-QTOF-MS	[151]
Paclitaxel and 5-fluorouracil	Paclitaxel was dissolved in methanol. The loading was performed by ultrasound treatment. The exosome surface was functionalized with activated folic acid	MCF-7 and MDA-MB-231 cells	Exosomes were stained with coumarin-6, the cell nuclei were stained with DAPI and observed under the fluorescence microscope	[152]
Paclitaxel	Exosomes were functionalized with folic acid. Exosome loading was performed by incubation	Xenografts of a subcutaneous lung tumor	Detection of orthotopic lung cancer using bioluminescent cells A549-Red-luc	[108]
**-**	**-**	Balb/c mice were inoculated with murine colon carcinoma cells (CT26)	Exosomes, labeled with DIR, SYTO 13 + DiD. Visualization by laser scanning confocal microscopy. Organs were imaged on Odyssey Imager	[29]
MicroRNA	miR-29b и miR-200c	Caco-2 and IEC-6 cells	Exosomes were labeled with fluorophore FM 4-64. Exosome uptake was analyzed by fluorescence on a Biotek FLx800 reader	[43]
Single-stranded RNA labeled with Exo-Glow Red	**-**	DiR-labeled bovine milk exosomes, administered to Balb/c mice via an oral gastric tube or intravenously	Fluorescence intensity was assessed by iBox or LI-COR Odyssey^®^ imaging systems	[32]
siRNA	Electroporation and chemical transfection	Lung cancer cell xenograft A549	Confocal microscopy of labeled siRNA	[92]
hsa-miR-148a-3p	Loading with lipofectamine	HepG2 cell lines, Caco-2	RT-PCR and qPCR	[101]
miR-31-5p	Electroporation	Mouse model of diabetic wound	Exosomes were stained with Dil, uptake was observed with laser scanning confocal microscope	[102]
Doxorubicin	Conjugation with milk exosomes via = CN bond	Intravenous administration to thymus-deprived mice carrying HSC-3, SCC-9 and CAL-27 cancer cells	Dox uptake was observed by confocal microscopy	[113]

## References

[1] Van Niel G., D’Angelo G., Raposo G. (2018). Shedding Light on the Cell Biology of Extracellular Vesicles. Nat. Rev. Mol. Cell Biol..

[2] Colombo M., Raposo G., Théry C. (2014). Biogenesis, Secretion, and Intercellular Interactions of Exosomes and Other Extracellular Vesicles. Annu. Rev. Cell Dev. Biol..

[3] Kalluri R., LeBleu V.S. (2020). The Biology, Function, and Biomedical Applications of Exosomes. Science.

[4] Tkach M., Théry C. (2016). Communication by Extracellular Vesicles: Where We Are and Where We Need to Go. Cell.

[5] Simpson R.J., Lim J.W., Moritz R.L., Mathivanan S. (2009). Exosomes: Proteomic Insights and Diagnostic Potential. Expert Rev. Proteom..

[6] Liang Y., Duan L., Lu J., Xia J. (2021). Engineering Exosomes for Targeted Drug Delivery. Theranostics.

[7] Pirisinu M., Pham T.C., Zhang D.X., Hong T.N., Nguyen L.T., Le M.T. (2022). Extracellular Vesicles as Natural Therapeutic Agents and Innate Drug Delivery Systems for Cancer Treatment: Recent Advances, Current Obstacles, and Challenges for Clinical Translation. Semin. Cancer Biol..

[8] Herrmann I.K., Wood M.J.A., Fuhrmann G. (2021). Extracellular Vesicles as a Next-Generation Drug Delivery Platform. Nat. Nanotechnol..

[9] Ullah M., Kodam S.P., Mu Q., Akbar A. (2021). Microbubbles versus Extracellular Vesicles as Therapeutic Cargo for Targeting Drug Delivery. ACS Nano.

[10] Yao X., Lyu P., Yoo K., Yadav M.K., Singh R., Atala A., Lu B. (2021). Engineered Extracellular Vesicles as Versatile Ribonucleoprotein Delivery Vehicles for Efficient and Safe CRISPR Genome Editing. J. Extracell. Vesicles.

[11] Sedykh S., Kuleshova A., Nevinsky G. (2020). Milk Exosomes: Perspective Agents for Anticancer Drug Delivery. Int. J. Mol. Sci..

[12] Alvarez-Erviti L., Seow Y., Yin H., Betts C., Lakhal S., Wood M.J.A. (2011). Delivery of SiRNA to the Mouse Brain by Systemic Injection of Targeted Exosomes. Nat. Biotechnol..

[13] Banks W.A., Sharma P., Bullock K.M., Hansen K.M., Ludwig N., Whiteside T.L. (2020). Transport of Extracellular Vesicles across the Blood-Brain Barrier: Brain Pharmacokinetics and Effects of Inflammation. Int. J. Mol. Sci..

[14] Record M. (2014). Intercellular Communication by Exosomes in Placenta: A Possible Role in Cell Fusion?. Placenta.

[15] Lee B., Saadeldin I., Oh H.J. (2015). Embryonic–Maternal Cross-Talk via Exosomes: Potential Implications. Stem Cells Cloning Adv. Appl..

[16] Haney M.J., Klyachko N.L., Zhao Y., Gupta R., Plotnikova E.G., He Z., Patel T., Piroyan A., Sokolsky M., Kabanov A.V. (2015). Exosomes as Drug Delivery Vehicles for Parkinson’s Disease Therapy. J. Control. Release.

[17] Kooijmans S.A.A., Gitz-Francois J.J.J.M., Schiffelers R.M., Vader P. (2018). Recombinant Phosphatidylserine-Binding Nanobodies for Targeting of Extracellular Vesicles to Tumor Cells: A Plug-and-Play Approach. Nanoscale.

[18] Antes T.J., Middleton R.C., Luther K.M., Ijichi T., Peck K.A., Liu W.J., Valle J., Echavez A.K., Marbán E. (2018). Targeting Extracellular Vesicles to Injured Tissue Using Membrane Cloaking and Surface Display. J. Nanobiotechnol..

[19] Whitford W., Guterstam P. (2019). Exosome Manufacturing Status. Future Med. Chem..

[20] Munagala R., Aqil F., Jeyabalan J., Gupta R.C. (2016). Bovine Milk-Derived Exosomes for Drug Delivery. Cancer Lett..

[21] Li Y., Xing L., Wang L., Liu X., Wu L., Ni M., Zhou Z., Li L., Liu X., Huang Y. (2023). Milk-Derived Exosomes as a Promising Vehicle for Oral Delivery of Hydrophilic Biomacromolecule Drugs. Asian J. Pharm. Sci..

[22] De la Torre Gomez C., Goreham R.V., Bech Serra J.J., Nann T., Kussmann M. (2018). “Exosomics”—A Review of Biophysics, Biology and Biochemistry of Exosomes with a Focus on Human Breast Milk. Front. Genet..

[23] Adriano B., Cotto N.M., Chauhan N., Jaggi M., Chauhan S.C., Yallapu M.M. (2021). Milk Exosomes: Nature’s Abundant Nanoplatform for Theranostic Applications. Bioact. Mater..

[24] Sedykh S.E., Burkova E.E., Purvinsh L.V., Klemeshova D.A., Ryabchikova E.I., Nevinsky G.A. (2020). Milk Exosomes: Isolation, Biochemistry, Morphology, and Perspectives of Use. Extracellular Vesicles and Their Importance in Human Health.

[25] Sedykh S.E., Purvinsh L.V., Burkova E.E., Dmitrenok P.S., Ryabchikova E.I., Nevinsky G.A. (2022). Analysis of Proteins and Peptides of Highly Purified CD9+ and CD63+ Horse Milk Exosomes Isolated by Affinity Chromatography. Int. J. Mol. Sci..

[26] Sedykh S.E., Purvinish L.V., Burkova E.E., Dmitrenok P.S., Vlassov V.V., Ryabchikova E.I., Nevinsky G.A. (2021). Analysis of Peptides and Small Proteins in Preparations of Horse Milk Exosomes, Purified on Anti-CD81-Sepharose. Int. Dairy J..

[27] Admyre C., Johansson S.M., Qazi K.R., Filén J.-J., Lahesmaa R., Norman M., Neve E.P.A., Scheynius A., Gabrielsson S. (2007). Exosomes with Immune Modulatory Features Are Present in Human Breast Milk. J. Immunol..

[28] Plantz P.E., Patton S., Keenan T.W. (1973). Further Evidence of Plasma Membrane Material in Skim Milk. J. Dairy Sci..

[29] Betker J.L., Angle B.M., Graner M.W., Anchordoquy T.J. (2019). The Potential of Exosomes from Cow Milk for Oral Delivery. J. Pharm. Sci..

[30] Somiya M., Yoshioka Y., Ochiya T. (2018). Biocompatibility of Highly Purified Bovine Milk-derived Extracellular Vesicles. J. Extracell. Vesicles.

[31] Sadri M., Shu J., Kachman S.D., Cui J., Zempleni J. (2020). Milk Exosomes and MiRNA Cross the Placenta and Promote Embryo Survival in Mice. Reproduction.

[32] Manca S., Upadhyaya B., Mutai E., Desaulniers A.T., Cederberg R.A., White B.R., Zempleni J. (2018). Milk Exosomes Are Bioavailable and Distinct MicroRNA Cargos Have Unique Tissue Distribution Patterns. Sci. Rep..

[33] Halvaei S., Daryani S., Eslami-S. Z., Samadi T., Jafarbeik-Iravani N., Bakhshayesh T.O., Majidzadeh-A. K., Esmaeili R. (2018). Exosomes in Cancer Liquid Biopsy: A Focus on Breast Cancer. Mol. Ther. Nucleic Acids.

[34] Qin W., Tsukasaki Y., Dasgupta S., Mukhopadhyay N., Ikebe M., Sauter E.R. (2016). Exosomes in Human Breast Milk Promote EMT. Clin. Cancer Res..

[35] Stefanon B., Cintio M., Sgorlon S., Scarsella E., Licastro D., Zecconi A., Colitti M. (2023). Regulatory Role of MicroRNA of Milk Exosomes in Mastitis of Dairy Cows. Animals.

[36] Reinhardt T.A., Sacco R.E., Nonnecke B.J., Lippolis J.D. (2013). Bovine Milk Proteome: Quantitative Changes in Normal Milk Exosomes, Milk Fat Globule Membranes and Whey Proteomes Resulting from Staphylococcus Aureus Mastitis. J. Proteom..

[37] Helewa M., Lévesque P., Provencher D., Lea R.H., Rosolowich V., Shapiro H.M., Breast Disease Committee and Executive Committeee and Council, Society of Obstetricians and Gynaecologists of Canada (2002). Breast Cancer, Pregnancy, and Breastfeeding. J. Obstet. Gynaecol. Can..

[38] Ahadian S., Finbloom J.A., Mofidfar M., Diltemiz S.E., Nasrollahi F., Davoodi E., Hosseini V., Mylonaki I., Sangabathuni S., Montazerian H. (2020). Micro and Nanoscale Technologies in Oral Drug Delivery. Adv. Drug Deliv. Rev..

[39] Bakhru S.H., Furtado S., Morello A.P., Mathiowitz E. (2013). Oral Delivery of Proteins by Biodegradable Nanoparticles. Adv. Drug Deliv. Rev..

[40] Liao Y., Du X., Li J., Lönnerdal B. (2017). Human Milk Exosomes and Their MicroRNAs Survive Digestion In Vitro and Are Taken Up by Human Intestinal Cells. Mol. Nutr. Food Res..

[41] Rani P., Vashisht M., Golla N., Shandilya S., Onteru S.K., Singh D. (2017). Milk MiRNAs Encapsulated in Exosomes Are Stable to Human Digestion and Permeable to Intestinal Barrier In Vitro. J. Funct. Foods.

[42] Kusuma R.J., Manca S., Friemel T., Sukreet S., Nguyen C., Zempleni J. (2016). Human Vascular Endothelial Cells Transport Foreign Exosomes from Cow’s Milk by Endocytosis. Am. J. Physiol. Physiol..

[43] Wolf T., Baier S.R., Zempleni J. (2015). The Intestinal Transport of Bovine Milk Exosomes Is Mediated by Endocytosis in Human Colon Carcinoma Caco-2 Cells and Rat Small Intestinal IEC-6 Cells. J. Nutr..

[44] Benmoussa A., Lee C.H.C., Laffont B., Savard P., Laugier J., Boilard E., Gilbert C., Fliss I., Provost P. (2016). Commercial Dairy Cow Milk MicroRNAs Resist Digestion under Simulated Gastrointestinal Tract Conditions. J. Nutr..

[45] Izumi H., Kosaka N., Shimizu T., Sekine K., Ochiya T., Takase M. (2012). Bovine Milk Contains MicroRNA and Messenger RNA That Are Stable under Degradative Conditions. J. Dairy Sci..

[46] Samuel M., Fonseka P., Sanwlani R., Gangoda L., Chee S.H., Keerthikumar S., Spurling A., Chitti S.V., Zanker D., Ang C.-S. (2021). Oral Administration of Bovine Milk-Derived Extracellular Vesicles Induces Senescence in the Primary Tumor but Accelerates Cancer Metastasis. Nat. Commun..

[47] Almeida J.P.M., Chen A.L., Foster A., Drezek R. (2011). In Vivo Biodistribution of Nanoparticles. Nanomedicine.

[48] González M.I., González-Arjona M., Santos-Coquillat A., Vaquero J., Vázquez-Ogando E., de Molina A., Peinado H., Desco M., Salinas B. (2021). Covalently Labeled Fluorescent Exosomes for In Vitro and In Vivo Applications. Biomedicines.

[49] González M.I., Martín-Duque P., Desco M., Salinas B. (2020). Radioactive Labeling of Milk-Derived Exosomes with 99mTc and In Vivo Tracking by SPECT Imaging. Nanomaterials.

[50] Witwer K.W., Buzás E.I., Bemis L.T., Bora A., Lässer C., Lötvall J., Nolte‘t Hoen E.N., Piper M.G., Sivaraman S., Skog J. (2013). Standardization of Sample Collection, Isolation and Analysis Methods in Extracellular Vesicle Research. J. Extracell. Vesicles.

[51] Buschmann D., Mussack V., Byrd J.B. (2021). Separation, Characterization, and Standardization of Extracellular Vesicles for Drug Delivery Applications. Adv. Drug Deliv. Rev..

[52] Vaswani K., Koh Y.Q., Almughlliq F.B., Peiris H.N., Mitchell M.D. (2017). A Method for the Isolation and Enrichment of Purified Bovine Milk Exosomes. Reprod. Biol..

[53] Samuel M., Chisanga D., Liem M., Keerthikumar S., Anand S., Ang C.-S., Adda C.G., Versteegen E., Jois M., Mathivanan S. (2017). Bovine Milk-Derived Exosomes from Colostrum Are Enriched with Proteins Implicated in Immune Response and Growth. Sci. Rep..

[54] Sukreet S., Braga C.P., An T.T., Adamec J., Cui J., Trible B., Zempleni J. (2021). Isolation of Extracellular Vesicles from Byproducts of Cheesemaking by Tangential Flow Filtration Yields Heterogeneous Fractions of Nanoparticles. J. Dairy Sci..

[55] Raju D., Bathini S., Badilescu S., Ghosh A., Packirisamy M. (2022). Microfluidic Platforms for the Isolation and Detection of Exosomes: A Brief Review. Micromachines.

[56] Wijenayake S., Eisha S., Tawhidi Z., Pitino M.A., Steele M.A., Fleming A.S., McGowan P.O. (2021). Comparison of Methods for Pre-Processing, Exosome Isolation, and RNA Extraction in Unpasteurized Bovine and Human Milk. PLoS ONE.

[57] Théry C., Witwer K.W., Aikawa E., Alcaraz M.J., Anderson J.D., Andriantsitohaina R., Antoniou A., Arab T., Archer F., Atkin-Smith G.K. (2018). Minimal Information for Studies of Extracellular Vesicles 2018 (MISEV2018): A Position Statement of the International Society for Extracellular Vesicles and Update of the MISEV2014 Guidelines. J. Extracell. Vesicles.

[58] Théry C., Amigorena S., Raposo G., Clayton A. (2006). Isolation and Characterization of Exosomes from Cell Culture Supernatants and Biological Fluids. Curr. Protoc. Cell Biol..

[59] Szatanek R., Baran J., Siedlar M., Baj-Krzyworzeka M. (2015). Isolation of Extracellular Vesicles: Determining the Correct Approach (Review). Int. J. Mol. Med..

[60] Sedykh S.E., Purvinish L.V., Monogarov A.S., Burkova E.E., Grigor’eva A.E., Bulgakov D.V., Dmitrenok P.S., Vlassov V.V., Ryabchikova E.I., Nevinsky G.A. (2017). Purified Horse Milk Exosomes Contain an Unpredictable Small Number of Major Proteins. Biochim. Open.

[61] Lönnerdal B. (2003). Nutritional and Physiologic Significance of Human Milk Proteins. Am. J. Clin. Nutr..

[62] Yamauchi M., Shimizu K., Rahman M., Ishikawa H., Takase H., Ugawa S., Okada A., Inoshima Y. (2019). Efficient Method for Isolation of Exosomes from Raw Bovine Milk. Drug Dev. Ind. Pharm..

[63] Rahman M.M., Shimizu K., Yamauchi M., Takase H., Ugawa S., Okada A., Inoshima Y. (2019). Acidification Effects on Isolation of Extracellular Vesicles from Bovine Milk. PLoS ONE.

[64] Li B., Hock A., Wu R.Y., Minich A., Botts S.R., Lee C., Antounians L., Miyake H., Koike Y., Chen Y. (2019). Bovine Milk-Derived Exosomes Enhance Goblet Cell Activity and Prevent the Development of Experimental Necrotizing Enterocolitis. PLoS ONE.

[65] Vaswani K., Mitchell M.D., Holland O.J., Qin Koh Y., Hill R.J., Harb T., Davies P.S.W., Peiris H. (2019). A Method for the Isolation of Exosomes from Human and Bovine Milk. J. Nutr. Metab..

[66] Ross M., Atalla H., Karrow N., Mallard B.A. (2021). The Bioactivity of Colostrum and Milk Exosomes of High, Average, and Low Immune Responder Cows on Human Intestinal Epithelial Cells. J. Dairy Sci..

[67] Yoo C.E., Kim G., Kim M., Park D., Kang H.J., Lee M., Huh N. (2012). A Direct Extraction Method for MicroRNAs from Exosomes Captured by Immunoaffinity Beads. Anal. Biochem..

[68] Shao H., Im H., Castro C.M., Breakefield X., Weissleder R., Lee H. (2018). New Technologies for Analysis of Extracellular Vesicles. Chem. Rev..

[69] Liga A., Vliegenthart A.D.B., Oosthuyzen W., Dear J.W., Kersaudy-Kerhoas M. (2015). Exosome Isolation: A Microfluidic Road-Map. Lab Chip.

[70] Li X., Su L., Zhang X., Chen Q., Wang Y., Shen Z., Zhong T., Wang L., Xiao Y., Feng X. (2022). Recent Advances on the Function and Purification of Milk Exosomes: A Review. Front. Nutr..

[71] Chen C., Skog J., Hsu C.-H., Lessard R.T., Balaj L., Wurdinger T., Carter B.S., Breakefield X.O., Toner M., Irimia D. (2010). Microfluidic Isolation and Transcriptome Analysis of Serum Microvesicles. Lab Chip.

[72] Wang Z., Wu H., Fine D., Schmulen J., Hu Y., Godin B., Zhang J.X.J., Liu X. (2013). Ciliated Micropillars for the Microfluidic-Based Isolation of Nanoscale Lipid Vesicles. Lab Chip.

[73] Yamada T., Inoshima Y., Matsuda T., Ishiguro N. (2012). Comparison of Methods for Isolating Exosomes from Bovine Milk. J. Vet. Med. Sci..

[74] Yamashita T., Takahashi Y., Nishikawa M., Takakura Y. (2016). Effect of Exosome Isolation Methods on Physicochemical Properties of Exosomes and Clearance of Exosomes from the Blood Circulation. Eur. J. Pharm. Biopharm..

[75] Doyle L., Wang M. (2019). Overview of Extracellular Vesicles, Their Origin, Composition, Purpose, and Methods for Exosome Isolation and Analysis. Cells.

[76] Van Der Pol E., Van Gemert M.J.C., Sturk A., Nieuwland R., Van Leeuwen T.G. (2012). Single vs. Swarm Detection of Microparticles and Exosomes by Flow Cytometry. J. Thromb. Haemost..

[77] Van der Vlist E.J., Nolte’t Hoen E.N.M., Stoorvogel W., Arkesteijn G.J.A., Wauben M.H.M. (2012). Fluorescent Labeling of Nano-Sized Vesicles Released by Cells and Subsequent Quantitative and Qualitative Analysis by High-Resolution Flow Cytometry. Nat. Protoc..

[78] Van der Pol E., Coumans F., Varga Z., Krumrey M., Nieuwland R. (2013). Innovation in Detection of Microparticles and Exosomes. J. Thromb. Haemost..

[79] Buzás E.I., Gardiner C., Lee C., Smith Z.J. (2017). Single Particle Analysis: Methods for Detection of Platelet Extracellular Vesicles in Suspension (Excluding Flow Cytometry). Platelets.

[80] Dragovic R.A., Gardiner C., Brooks A.S., Tannetta D.S., Ferguson D.J.P., Hole P., Carr B., Redman C.W.G., Harris A.L., Dobson P.J. (2011). Sizing and Phenotyping of Cellular Vesicles Using Nanoparticle Tracking Analysis. Nanomed. Nanotechnol. Biol. Med..

[81] Raval N., Maheshwari R., Kalyane D., Youngren-Ortiz S.R., Chougule M.B., Tekade R.K. (2019). Importance of Physicochemical Characterization of Nanoparticles in Pharmaceutical Product Development. Basic Fundamentals of Drug Delivery.

[82] Kovrigina E., Chubarov A., Dmitrienko E. (2022). High Drug Capacity Doxorubicin-Loaded Iron Oxide Nanocomposites for Cancer Therapy. Magnetochemistry.

[83] Shim M.K., Park J., Yoon H.Y., Lee S., Um W., Kim J.-H., Kang S.-W., Seo J.-W., Hyun S.-W., Park J.H. (2019). Carrier-Free Nanoparticles of Cathepsin B-Cleavable Peptide-Conjugated Doxorubicin Prodrug for Cancer Targeting Therapy. J. Control. Release.

[84] Tutanov O., Orlova E., Proskura K., Grigor’eva A., Yunusova N., Tsentalovich Y., Alexandrova A., Tamkovich S. (2020). Proteomic Analysis of Blood Exosomes from Healthy Females and Breast Cancer Patients Reveals an Association between Different Exosomal Bioactivity on Non-Tumorigenic Epithelial Cell and Breast Cancer Cell Migration in Vitro. Biomolecules.

[85] Colitti M., Sgorlon S., Licastro D., Stefanon B. (2019). Differential Expression of MiRNAs in Milk Exosomes of Cows Subjected to Group Relocation. Res. Vet. Sci..

[86] Raposo G., Stoorvogel W. (2013). Extracellular Vesicles: Exosomes, Microvesicles, and Friends. J. Cell Biol..

[87] Pols M.S., Klumperman J. (2009). Trafficking and Function of the Tetraspanin CD63. Exp. Cell Res..

[88] Wang X., Tian F., Chen C., Feng Y., Sheng X., Guo Y., Ni H. (2019). Exosome-Derived Uterine MicroRNAs Isolated from Cows with Endometritis Impede Blastocyst Development. Reprod. Biol..

[89] Blans K., Hansen M.S., Sørensen L.V., Hvam M.L., Howard K.A., Möller A., Wiking L., Larsen L.B., Rasmussen J.T. (2017). Pellet-Free Isolation of Human and Bovine Milk Extracellular Vesicles by Size-Exclusion Chromatography. J. Extracell. Vesicles.

[90] Shtam T.A., Kovalev R.A., Varfolomeeva E.Y., Makarov E.M., Kil Y.V., Filatov M.V. (2013). Exosomes Are Natural Carriers of Exogenous SiRNA to Human Cells in Vitro. Cell Commun. Signal..

[91] Wahlgren J., Karlson T.D.L., Brisslert M., Vaziri Sani F., Telemo E., Sunnerhagen P., Valadi H. (2012). Plasma Exosomes Can Deliver Exogenous Short Interfering RNA to Monocytes and Lymphocytes. Nucleic Acids Res..

[92] Aqil F., Munagala R., Jeyabalan J., Agrawal A.K., Kyakulaga A.-H., Wilcher S.A., Gupta R.C. (2019). Milk Exosomes—Natural Nanoparticles for SiRNA Delivery. Cancer Lett..

[93] Xiang X., Chen J., Jiang T., Yan C., Kang Y., Zhang M., Xiang K., Guo J., Jiang G., Wang C. (2023). Milk-Derived Exosomes Carrying SiRNA-KEAP1 Promote Diabetic Wound Healing by Improving Oxidative Stress. Drug Deliv. Transl. Res..

[94] Warren M.R., Zhang C., Vedadghavami A., Bokvist K., Dhal P.K., Bajpayee A.G. (2021). Milk Exosomes with Enhanced Mucus Penetrability for Oral Delivery of SiRNA. Biomater. Sci..

[95] Ambros V. (2001). MicroRNAs. Cell.

[96] Friedman R.C., Farh K.K.-H., Burge C.B., Bartel D.P. (2009). Most Mammalian MRNAs Are Conserved Targets of MicroRNAs. Genome Res..

[97] Bernstein E., Kim S.Y., Carmell M.A., Murchison E.P., Alcorn H., Li M.Z., Mills A.A., Elledge S.J., Anderson K.V., Hannon G.J. (2003). Dicer Is Essential for Mouse Development. Nat. Genet..

[98] Lu J., Clark A.G. (2012). Impact of MicroRNA Regulation on Variation in Human Gene Expression. Genome Res..

[99] Boca S., Gulei D., Zimta A.-A., Onaciu A., Magdo L., Tigu A.B., Ionescu C., Irimie A., Buiga R., Berindan-Neagoe I. (2020). Nanoscale Delivery Systems for MicroRNAs in Cancer Therapy. Cell. Mol. Life Sci..

[100] Meng Z., Zhou D., Gao Y., Zeng M., Wang W. (2018). MiRNA Delivery for Skin Wound Healing. Adv. Drug Deliv. Rev..

[101] Del Pozo-Acebo L., López de las Hazas M.-C., Tomé-Carneiro J., Gil-Cabrerizo P., San-Cristobal R., Busto R., García-Ruiz A., Dávalos A. (2021). Bovine Milk-Derived Exosomes as a Drug Delivery Vehicle for MiRNA-Based Therapy. Int. J. Mol. Sci..

[102] Yan C., Chen J., Wang C., Yuan M., Kang Y., Wu Z., Li W., Zhang G., Machens H.-G., Rinkevich Y. (2022). Milk Exosomes-Mediated MiR-31-5p Delivery Accelerates Diabetic Wound Healing through Promoting Angiogenesis. Drug Deliv..

[103] Tao H., Xu H., Zuo L., Li C., Qiao G., Guo M., Zheng L., Leitgeb M., Lin X. (2020). Exosomes-Coated Bcl-2 SiRNA Inhibits the Growth of Digestive System Tumors Both in Vitro and in Vivo. Int. J. Biol. Macromol..

[104] Vashisht M., Rani P., Onteru S.K., Singh D. (2017). Curcumin Encapsulated in Milk Exosomes Resists Human Digestion and Possesses Enhanced Intestinal Permeability In Vitro. Appl. Biochem. Biotechnol..

[105] Jiang Q.-W., Cheng K.-J., Mei X.-L., Qiu J.-G., Zhang W.-J., Xue Y.-Q., Qin W.-M., Yang Y., Zheng D.-W., Chen Y. (2015). Synergistic Anticancer Effects of Triptolide and Celastrol, Two Main Compounds from Thunder God Vine. Oncotarget.

[106] Li Z., Wu X., Li J., Yao L., Sun L., Shi Y., Zhang W., Lin J., Liang D., Li Y. (2012). Antitumor Activity of Celastrol Nanoparticles in a Xenograft Retinoblastoma Tumor Model. Int. J. Nanomed..

[107] Aqil F., Kausar H., Agrawal A.K., Jeyabalan J., Kyakulaga A.-H., Munagala R., Gupta R. (2016). Exosomal Formulation Enhances Therapeutic Response of Celastrol against Lung Cancer. Exp. Mol. Pathol..

[108] Kandimalla R., Aqil F., Alhakeem S.S., Jeyabalan J., Tyagi N., Agrawal A., Yan J., Spencer W., Bondada S., Gupta R.C. (2021). Targeted Oral Delivery of Paclitaxel Using Colostrum-Derived Exosomes. Cancers.

[109] Agrawal A.K., Aqil F., Jeyabalan J., Spencer W.A., Beck J., Gachuki B.W., Alhakeem S.S., Oben K., Munagala R., Bondada S. (2017). Milk-Derived Exosomes for Oral Delivery of Paclitaxel. Nanomed. Nanotechnol. Biol. Med..

[110] Iqbal N., Iqbal N. (2014). Human Epidermal Growth Factor Receptor 2 (HER2) in Cancers: Overexpression and Therapeutic Implications. Mol. Biol. Int..

[111] Ewer M.S., Ewer S.M. (2015). Erratum: Cardiotoxicity of Anticancer Treatments. Nat. Rev. Cardiol..

[112] Pullan J., Dailey K., Bhallamudi S., Feng L., Alhalhooly L., Froberg J., Osborn J., Sarkar K., Molden T., Sathish V. (2022). Modified Bovine Milk Exosomes for Doxorubicin Delivery to Triple-Negative Breast Cancer Cells. ACS Appl. Bio Mater..

[113] Zhang Q., Xiao Q., Yin H., Xia C., Pu Y., He Z., Hu Q., Wang J., Wang Y. (2020). Milk-Exosome Based PH/Light Sensitive Drug System to Enhance Anticancer Activity against Oral Squamous Cell Carcinoma. RSC Adv..

[114] Wang L., Shi Z., Wang X., Mu S., Xu X., Shen L., Li P. (2021). Protective Effects of Bovine Milk Exosomes against Oxidative Stress in IEC-6 Cells. Eur. J. Nutr..

[115] Onizuka Y., Fujita K., Ide S., Naito T., Kaji N. (2023). Antioxidants Encapsulated Milk-Derived Exosomes for Functional Food Development. Anal. Sci..

[116] Fu S., Wang Y., Xia X., Zheng J.C. (2020). Exosome Engineering: Current Progress in Cargo Loading and Targeted Delivery. Nano Impact.

[117] Sun D., Zhuang X., Xiang X., Liu Y., Zhang S., Liu C., Barnes S., Grizzle W., Miller D., Zhang H.-G. (2010). A Novel Nanoparticle Drug Delivery System: The Anti-Inflammatory Activity of Curcumin Is Enhanced When Encapsulated in Exosomes. Mol. Ther..

[118] Yang T., Martin P., Fogarty B., Brown A., Schurman K., Phipps R., Yin V.P., Lockman P., Bai S. (2015). Exosome Delivered Anticancer Drugs Across the Blood-Brain Barrier for Brain Cancer Therapy in Danio Rerio. Pharm. Res..

[119] Munagala R., Aqil F., Jeyabalan J., Agrawal A.K., Mudd A.M., Kyakulaga A.H., Singh I.P., Vadhanam M.V., Gupta R.C. (2017). Exosomal Formulation of Anthocyanidins against Multiple Cancer Types. Cancer Lett..

[120] Shi Y., Guo S., Liang Y., Liu L., Wang A., Sun K., Li Y. (2022). Construction and Evaluation of Liraglutide Delivery System Based on Milk Exosomes: A New Idea for Oral Peptide Delivery. Curr. Pharm. Biotechnol..

[121] Asadirad A., Hashemi S.M., Baghaei K., Ghanbarian H., Mortaz E., Zali M.R., Amani D. (2019). Phenotypical and Functional Evaluation of Dendritic Cells after Exosomal Delivery of MiRNA-155. Life Sci..

[122] Johnsen K.B., Gudbergsson J.M., Skov M.N., Christiansen G., Gurevich L., Moos T., Duroux M. (2016). Evaluation of Electroporation-Induced Adverse Effects on Adipose-Derived Stem Cell Exosomes. Cytotechnology.

[123] Hood J.L., Scott M.J., Wickline S.A. (2014). Maximizing Exosome Colloidal Stability Following Electroporation. Anal. Biochem..

[124] Mukhopadhya A., Tsiapalis D., McNamee N., Talbot B., O’Driscoll L. (2023). Doxorubicin Loading into Milk and Mesenchymal Stem Cells’ Extracellular Vesicles as Drug Delivery Vehicles. Pharmaceutics.

[125] Kim M.S., Haney M.J., Zhao Y., Mahajan V., Deygen I., Klyachko N.L., Inskoe E., Piroyan A., Sokolsky M., Okolie O. (2016). Development of Exosome-Encapsulated Paclitaxel to Overcome MDR in Cancer Cells. Nanomed. Nanotechnol. Biol. Med..

[126] Sato Y.T., Umezaki K., Sawada S., Mukai S., Sasaki Y., Harada N., Shiku H., Akiyoshi K. (2016). Engineering Hybrid Exosomes by Membrane Fusion with Liposomes. Sci. Rep..

[127] Podolak I., Galanty A., Sobolewska D. (2010). Saponins as Cytotoxic Agents: A Review. Phytochem. Rev..

[128] Fuhrmann G., Serio A., Mazo M., Nair R., Stevens M.M. (2015). Active Loading into Extracellular Vesicles Significantly Improves the Cellular Uptake and Photodynamic Effect of Porphyrins. J. Control. Release.

[129] Wiklander O.P.B., Nordin J.Z., O’Loughlin A., Gustafsson Y., Corso G., Mäger I., Vader P., Lee Y., Sork H., Seow Y. (2015). Extracellular Vesicle in Vivo Biodistribution Is Determined by Cell Source, Route of Administration and Targeting. J. Extracell. Vesicles.

[130] Feng X., Chen X., Zheng X., Zhu H., Qi Q., Liu S., Zhang H., Che J. (2021). Latest Trend of Milk Derived Exosomes: Cargos, Functions, and Applications. Front. Nutr..

[131] Wang Z., Shi H., Guo J., Li C. (2015). A Current Review of Folate Receptor Alpha as a Potential Tumor Target in Non-Small-Cell Lung Cancer. Drug Des. Dev. Ther..

[132] Cheung A., Bax H.J., Josephs D.H., Ilieva K.M., Pellizzari G., Opzoomer J., Bloomfield J., Fittall M., Grigoriadis A., Figini M. (2016). Targeting Folate Receptor Alpha for Cancer Treatment. Oncotarget.

[133] Kim K.-U., Kim W.-H., Jeong C.H., Yi D.Y., Min H. (2020). More than Nutrition: Therapeutic Potential of Breast Milk-Derived Exosomes in Cancer. Int. J. Mol. Sci..

[134] Naor D., Nedvetzki S., Golan I., Melnik L., Faitelson Y. (2002). CD44 in Cancer. Crit. Rev. Clin. Lab. Sci..

[135] Li D., Yao S., Zhou Z., Shi J., Huang Z., Wu Z. (2020). Hyaluronan Decoration of Milk Exosomes Directs Tumor-Specific Delivery of Doxorubicin. Carbohydr. Res..

[136] Counihan J.L., Grossman E.A., Nomura D.K. (2018). Cancer Metabolism: Current Understanding and Therapies. Chem. Rev..

[137] Liu S., Luo X., Liu S., Xu P., Wang J., Hu Y. (2019). Acetazolamide-Loaded PH-Responsive Nanoparticles Alleviating Tumor Acidosis to Enhance Chemotherapy Effects. Macromol. Biosci..

[138] Tian T., Zhang H.-X., He C.-P., Fan S., Zhu Y.-L., Qi C., Huang N.-P., Xiao Z.-D., Lu Z.-H., Tannous B.A. (2018). Surface Functionalized Exosomes as Targeted Drug Delivery Vehicles for Cerebral Ischemia Therapy. Biomaterials.

[139] Smyth T., Petrova K., Payton N.M., Persaud I., Redzic J.S., Graner M.W., Smith-Jones P., Anchordoquy T.J. (2014). Surface Functionalization of Exosomes Using Click Chemistry. Bioconjug. Chem..

[140] Kooijmans S.A.A., Fliervoet L.A.L., van der Meel R., Fens M.H.A.M., Heijnen H.F.G., van Bergen en Henegouwen P.M.P., Vader P., Schiffelers R.M. (2016). PEGylated and Targeted Extracellular Vesicles Display Enhanced Cell Specificity and Circulation Time. J. Control. Release.

[141] Kim M.S., Haney M.J., Zhao Y., Yuan D., Deygen I., Klyachko N.L., Kabanov A.V., Batrakova E.V. (2018). Engineering Macrophage-Derived Exosomes for Targeted Paclitaxel Delivery to Pulmonary Metastases: In Vitro and in Vivo Evaluations. Nanomed. Nanotechnol. Biol. Med..

[142] Cao Y., Wu T., Zhang K., Meng X., Dai W., Wang D., Dong H., Zhang X. (2019). Engineered Exosome-Mediated Near-Infrared-II Region V 2 C Quantum Dot Delivery for Nucleus-Target Low-Temperature Photothermal Therapy. ACS Nano.

[143] Pi F., Binzel D.W., Lee T.J., Li Z., Sun M., Rychahou P., Li H., Haque F., Wang S., Croce C.M. (2018). Nanoparticle Orientation to Control RNA Loading and Ligand Display on Extracellular Vesicles for Cancer Regression. Nat. Nanotechnol..

[144] Nag O., Awasthi V. (2013). Surface Engineering of Liposomes for Stealth Behavior. Pharmaceutics.

[145] Won Y.-W., Patel A.N., Bull D.A. (2014). Cell Surface Engineering to Enhance Mesenchymal Stem Cell Migration toward an SDF-1 Gradient. Biomaterials.

[146] Yi Y.W., Lee J.H., Kim S.-Y., Pack C.-G., Ha D.H., Park S.R., Youn J., Cho B.S. (2020). Advances in Analysis of Biodistribution of Exosomes by Molecular Imaging. Int. J. Mol. Sci..

[147] Jensen E.C. (2012). Use of Fluorescent Probes: Their Effect on Cell Biology and Limitations. Anat. Rec. Adv. Integr. Anat. Evol. Biol..

[148] Dias M.V.S., Martins V.R., Hajj G.N.M. (2016). Stress-Inducible Protein 1 (STI1): Extracellular Vesicle Analysis and Quantification. Unconventional Protein Secretion.

[149] Zhou G., Gu Y., Zhu Z., Zhang H., Liu W., Xu B., Zhou F., Zhang M., Hua K., Wu L. (2022). Exosome Mediated Cytosolic Cisplatin Delivery Through Clathrin-Independent Endocytosis and Enhanced Anti-Cancer Effect via Avoiding Endosome Trapping in Cisplatin-Resistant Ovarian Cancer. Front. Med..

[150] Wu L., Wang L., Liu X., Bai Y., Wu R., Li X., Mao Y., Zhang L., Zheng Y., Gong T. (2022). Milk-Derived Exosomes Exhibit Versatile Effects for Improved Oral Drug Delivery. Acta Pharm. Sin. B.

[151] González-Sarrías A., Iglesias-Aguirre C.E., Cortés-Martín A., Vallejo F., Cattivelli A., del Pozo-Acebo L., Del Saz A., López de las Hazas M.C., Dávalos A., Espín J.C. (2022). Milk-Derived Exosomes as Nanocarriers to Deliver Curcumin and Resveratrol in Breast Tissue and Enhance Their Anticancer Activity. Int. J. Mol. Sci..

[152] Kumar D.N., Chaudhuri A., Dehari D., Shekher A., Gupta S.C., Majumdar S., Krishnamurthy S., Singh S., Kumar D., Agrawal A.K. (2022). Combination Therapy Comprising Paclitaxel and 5-Fluorouracil by Using Folic Acid Functionalized Bovine Milk Exosomes Improves the Therapeutic Efficacy against Breast Cancer. Life.

